# The influence of acute hypoxic exposure on isokinetic muscle force production

**DOI:** 10.1186/2193-1801-3-604

**Published:** 2014-10-15

**Authors:** Rafael Kenji Ivamoto, Fernanda Patti Nakamoto, Rodrigo Luiz Vancini, Ana Amélia Benedito-Silva, Claudio Andre Barbosa de Lira, Marília dos Santos Andrade

**Affiliations:** Departamento de Fisiologia, Universidade Federal de São Paulo (UNIFESP), Rua Botucatu, 862, 5º andar, Ed. Ciências Biomédicas, CEP 04023-062 São Paulo, SP Brazil; Centro de Educação Física e Desportos (CEFD), Universidade Federal do Espírito Santo (UFES), Vitória, ES Brazil; Escola de Artes, Ciências e Humanidades, Universidade de São Paulo (USP), São Paulo, SP Brazil; Setor de Fisiologia Humana e do Exercício, Faculdade de Educação Física e Dança (FEFD), Universidade Federal de Goiás (UFG), Goiânia, GO Brazil

**Keywords:** Normoxia, Isokinetic dynamometry, Knee muscle strength, Oxygen saturation

## Abstract

To investigated whether an acute hypoxic stimulus affects muscle strength development assessed by isokinetic dynamometry during maximal knee extension. A total of 15 healthy young men participated in this study (61.9 ± 6.1 kg; 1.72 ± 0.08 m; 20.9 ± 2.6 years). We evaluated knee extension and flexion isokinetic dynamometer performance in normoxic and hypoxic conditions. The analyzed parameters, for concentric contraction, were peak torque and total work measured at 1.05 and 5.23 rad/s; and fatigue index measured at 5.23 rad/s. During isokinetic testing, heart rate and oxygen saturation (SpO_2_) were monitored. Hypoxic conditions (3,600 m) were simulated, via a mixing chamber, with the dilution being constantly controlled by a PO_2_ probe. Test reproducibility results (test-retest) for all isokinetic knee parameters were classified as moderate to almost perfect (ICC = 0.694 to 0.932). SpO_2_ was 88.4 ± 3.4% in the hypoxic condition and 97.1 ± 0.7% in the normoxic condition (p = 0.000, effect size = 0.87). Heart rate was not significantly different between normoxic and hypoxic conditions at the end of the test. There were no significant differences in isokinetic variables evaluated for the extensor and flexor muscles at concentric contraction between the normoxic and hypoxic conditions. Our findings indicate that reduced arterial oxygenation *per se* has no effect on the muscular isokinetic strength of the knee extensors.

## Introduction

It is well known that a hypoxemic stimulus produces a significant reduction in aerobic performance (Wehrlin and Hallen 2006), however less is known about the effects of hypoxemic exposure on muscle strength development. During chronic exposure to hypobaric hypoxemia (real or simulated high altitude), several authors reported no change in maximal voluntary contraction - MVC (Garner et al. 1990; Fulco et al. 1994; Orizio et al. 1994; Caquelard et al. 2000). By contrast, during acute hypoxemia (inhalation of 10 to 12% oxygen gas mixtures) the results are contradictory since several authors noted a decrease in MVC (Eiken and Tesch 1984; Badier et al. 1994; Bendahan et al. 1998). This has been attributed to alterations in muscle energetics, impaired neuromuscular transmission, and/or changes in the central drive during contractions.

Dousset et al. (2001) showed a decrease in MVC and a reduction of central drive to muscle fibers in hypoxic conditions. Also, a reduction in the recruitment of slow-twitch motor units (highly oxygen-dependent fibers) was observed. These data corroborate the Central Governor Theory proposed by Hill, Long and Lupton between 1923 and 1925 which is supported by Noakes et al. (2001). These authors proposed that peripheral fatigue occurs only after the onset of myocardial fatigue (it could also be due to respiratory muscle or central nervous system fatigue) that constantly send, by afferent fibers information to brain (the central governor) about peripheral metabolic status. If there is any threat of ischemia or hypoxia, the central governor immediately reduces efferent neural activation to the exercised muscles, thus decreasing the muscle mass that can be recruited (Noakes et al. 2001).

It is clear that the effects of acute hypoxia on muscular strength are a topic of extensive debate. Thus, the purpose of this study was to investigate whether an acute hypoxic stimulus affects muscle strength development assessed by isokinetic dynamometry during maximal isokinetic knee extension and flexion. Obtaining these responses is valuable for guidance training in hypoxic conditions.

## Methods

### Participants

A total of 15 young men volunteered to participate in this study (body mass: 61.9 ± 6.1 kg; height: 1.72 ± 0.08 m; age: 20.9 ± 2.6 years, data are presented as mean ± standard deviation). Participants were recruited among students of the Federal University of São Paulo (São Paulo, Brazil). The participants were sedentary or recreationally active and asymptomatic. The inclusion criteria were: not having participated in resistance training for at least a year before the study; able to perform physical exercise; not having been exposed to altitudes above 3000 m at least one month before study; no chronic disease; and not currently smoking or using drugs/ergogenic aids that could affect muscular function. After a clear explanation of the procedures, including the risks and benefits of participation, written consent was obtained. All experimental procedures were approved by the University Human Research Ethics Committee and they conformed to the principles outlined in the Declaration of Helsinki. All participants were highly motivated to participate in the study. They were requested to refrain from strenuous workouts the day before the experimental procedures.

### Study design

The study was organized in three successive phases: (1) familiarization with isokinetic dynamometer and isokinetic assessment in normoxic (2) or hypoxic condition (3). The order of phases (2) and (3) was randomized. These phases were separated by at least 1 day up to a maximum of 3 days. We correlated the isokinetic assessment results performed in phase 1 with the normoxic condition assessment results in order to verify the reproducibility of the tests. The hypoxic condition evaluation results were compared with the normoxic condition results in order to compare the effect of hypoxia on muscle strength development. Before isokinetic assessment, participants were submitted to 10 minutes under hypoxic stimulus.

### Isokinetic procedures

Before isokinetic assessment, a 5 minute warm-up was performed on a cycle ergometer (Schwinn Air-Dyne®, Nautilus Inc, USA) at a resistance level of 50 W. Following the warm-up period, subjects were placed in the isokinetic dynamometer (Cybex 6000, Ronkonkowa, New York, USA) to perform concentric strength exercise bouts for dominant lower limbs. Limb dominance was determined by asking the subjects which limb they preferred to use when kicking a ball. Subjects assumed a seated position on the isokinetic dynamometer with their hips flexed at approximately 85 degrees. Standard stabilization strapping was placed across the trunk and waist. The distal femur of the limb was also stabilized during the tests to minimize additional movement and to ensure identical conditions for all participants. The dynamometer axis was visually aligned with lateral femoral condyle while the knees were flexed at 90 degrees. The length of the lever arm was individually determined by the length of each individual’s lower leg, and the resistance pad was placed proximal to the medial malleolus. Following the direct measurement of the lower limb mass lever system at 30 degrees of knee extension, gravity correction procedures were applied according to the manufacturer’s specifications to reduce the risk of inaccurate data.

As part of the familiarization process, the participants were given standard verbal instructions regarding the procedures and were allowed several sub-maximal practice attempts for each angular velocity used (1.05 and 5.23 rad/s). The subjects were then tested with 5 repetitions at 1.05 rad/s and 30 repetitions at 5.23 rad/s performed at concentric contraction, and the results were stored for analysis. All subjects received a 60 second rest period between sets to prevent fatigue build-up. Verbal commands were given by the examiner before each test to ensure maximal effort, and visual feedback of the recorded torque was provided during the test. All subjects were tested by the same examiner who was trained and experienced in the use of isokinetic testing devices.

The evaluated parameters were: 1) hamstring and quadriceps isokinetic peak torque at 1.05 and 5.23 rad/s in Newton.m (Nm); 2) hamstring and quadriceps muscles total work in Joules (J) measured at 1.05 and 5.23 rad/s; and 3) fatigue index as a percentage measured at 5.23 rad/s. All obtained values correspond to concentric contractions. The fatigue index was calculated by dividing the minimum torque values by the maximum torque values at the same angular speed. In order to verify whether the participant actually performed maximal voluntary contraction in all repetitions of the test, we evaluated the variation coefficient provided by the equipment software (standard deviation expressed as a percentage of the mean). The maximum variation accepted was 10%. The dynamometer was monthly calibrated according to the manufacturer’s specifications and was checked prior to each individual test.

During isokinetic testing, heart rate (HR) and oxygen saturation (SpO_2_) were monitored. Heart rate registers (5 seconds average HR value) were monitored by a commercially available system (S810i, Polar Electronics, Kempele, Oulu) and SpO_2_ was monitored every minute by a pulse oximeter (Pulse Oximeter OctiveTech 300CSE®, Atlanta, USA).

### Altitude simulation

Normobaric hypoxic conditions corresponding to an altitude of 3,600 m (fraction of inspired oxygen - FIO_2_ = 13%) were simulated, according to Kon et al. (2010), by diluting ambient air with nitrogen (Air Liquide, São Paulo, Brazil) via a mixing chamber, with the dilution being constantly controlled by an oxygen partial pressure (PO_2_) probe (AltiTrainer200, Sport and Medical Technology, Geneva, Switzerland). This device allows the inspired PO_2_ to be set at a predetermined value to simulate altitude. Participants were attached to the device through a mask. The precision of the PO_2_ is of ±0.82 Torr. The device was calibrated weekly according to the manufacturer’s instructions. The hypoxic stimulus was initiated 10 minutes prior to isokinetic testing.

During the normobaric condition, participants underwent the same procedure as for the hypoxic condition, namely that they were coupled to the device through a mask, but instead breathed room air (FIO_2_ = 20.93%).

### Statistical analysis

A clinically important and relevant difference in peak torque value has been suggested to be 15% (Croisier et al. 2008, Yeung et al. 2009). A sample size calculation on the peak torque, using a logistic rank survival power analysis, showed that fifteen subjects are needed to detect a clinically relevant difference with 80% power and a significance level of 5%.

All variables presented normal distributions (p > 0.05) according to Kolmogorov-Smirnov tests. Intraclass correlation coefficients’ (ICCs) were calculated to determine the test-retest reliability. The ICCs were interpreted as follows: 0.90-0.99, almost perfect agreement; 0.70-0.89, strong agreement; 0.50-0.69, moderate agreement; 0.3-0.49, fair agreement; 0.0-0.29, poor agreement (Munro et al. 1986). Subsequently, the differences between the two conditions (normoxia versus hypoxia) were analyzed with a paired Student-*t* test. The level of significance was p < 0.05. Data are showed as mean ± standard deviation (SD). All statistical analyses were performed with Statistica, version 7.0 (Statsoft Inc, Tulsa, Oklahoma, USA). In addition, the measures of the effect size for changes in outcome were calculated by dividing the mean difference by the standard deviation (SD) of the normobaric hypoxic conditions measurement. Calculating effect sizes, the magnitude of any change was judged according to the following criteria: ES = 0.2 considered a “small” effect size; 0.5 represented a “medium” effect size; and 0.8 a “large” effect size (Cohen 1988).

### Results

The test-retest ICCs (test reproducibility results) for all measurements were classified as moderate to almost perfect as following: Extensor muscles peak torque at 5.23 rad/s - ICC = 0.927, almost perfect agreement; Extensor muscles peak torque at 1.05 rad/s - ICC = 0.866, strong agreement; Flexor muscles peak torque at 5.23 rad/s - ICC = 0.800, strong agreement; Flexor muscles peak torque 1.05 rad/s - ICC = 0.735, strong agreement; Extensor muscles total work at 5.23 rad/s - ICC = 0.694, moderate agreement; Flexor muscles total work at 5.23 rad/s - ICC = 0.821, strong agreement; Extensor muscles fatigue index (%) - ICC = 0.932, almost perfect agreement; and Flexor muscles fatigue index (%) - ICC = 0.798, strong agreement.

During all experimental procedures conducted in hypoxia conditions, SpO_2_ was 88.4 ± 3.4% and in normoxia conditions was 97.1 ± 0.7%. This demonstrates that there were significant differences between conditions (p = 0.000, d = 3.54). Heart rate was not significantly different between normoxic and hypoxic conditions at the end of the test (156 ± 23 bpm and 158 ± 16 bpm, respectively; p = 0.41, d = 0.10).

Table 1 presents the isokinetic performance of extensor and flexor muscles at concentric contraction during normoxia and hypoxia conditions. There were no significant differences between isokinetic variables evaluated in both conditions (p > 0.05).

**Table 1:** **Isokinetic muscle performance at concentric contraction in normoxia and hypoxia conditions**

Angular velocity (rad/s)	Isokinetic measure	Muscle group	Normoxia	Hypoxia	Effect size (Cohen’s d)
**1.05**	**Peak torque (Nm)**	*Extensors*	181.2 ± 31.5	178.9 ± 31.8	0.07
		*Flexors*	107.5 ± 12.6	107.9 ± 13.4	0.03
	**Total work (Joules)**	*Extensors*	185.3 ± 33.4	186.7 ± 38.9	0.04
		*Flexors*	120.9 ± 19.2	123.3 ± 25.3	0.10
**5.23**	**Peak torque (Nm)**	*Extensors*	100.7 ± 10.8	105.3 ± 18.3	0.30
		*Flexors*	70.8 ± 13.2	74.7 ± 17.1	0.25
	**Total work (Joules)**	*Extensors*	1619.7 ± 324.5	1696.2 ± 454.8	0.19
		*Flexors*	941.7 ± 257.5	948.0 ± 304.5	0.02
	**Fatigue index (%)**	*Extensors*	32.3 ± 31.7	35.8 ± 12.7	0.15
		*Flexors*	30.93 ± 35.04	42.13 ± 13.74	0.42

Data are presented as mean ± SD.

## Discussion

Hypoxia is responsible for a number of physiological adaptations that affect performance (Ortega et al. 2004; Sharp and Bernaudin 2004). However, there is no consensus in the literature on the effects of an acute hypoxic stimulus on the development of muscle torque and muscle fatigue. For this reason, the present study assessed the effects of normoxic and hypoxic conditions on muscular isokinetic torque production and resistance to fatigue. Our main finding was that, although the SpO_2_ level was lower in the hypoxic condition, the peak torque and total work values did not differ between the two conditions. Our data suggest that muscular isokinetic strength and resistance to fatigue were not influenced by acute hypoxia and support existing evidence that hypoxia has a minimal effect on voluntary force (Amann and Kayser 2009; Perrey and Rupp 2009).

Resistance to fatigue has been shown to be lower during whole body exercise under hypoxemic conditions, however the fatigue mechanism during whole body exercise is complex and multifactorial there is not only a decrease in SpO_2_, but also an increase in cardiorespiratory requirements. When a small muscle mass is activated, on the other hand, a given absolute force output is exerted and the cardiorespiratory requirements are reduced (Calbet et al. 2009). Thus small muscle mass is a suitable model for investigating the independent effects of SpO_2_ on muscle fatigue. Our results show no significant difference (normoxia versus hypoxia) in fatigue index related to isokinetic peak torque and total work. This result is comparable with data from other studies showing that FIO_2_ has little effect on fatigue (Eiken and Tesch 1984; McLellan et al. 1990; Calbet et al. 2003).

The fact that muscular strength is not reduced during acute moderate hypoxemia does not therefore confirm previous data (Eiken and Tesch 1984; Badier et al. 1994) nor the hypothesis that hypoxia-induced metabolic disturbance evokes a rise in discharge frequency of group III/IV muscle afferents (Hill et al. 1992) that may affect muscular strength or muscular fatigue through increased inhibitory influence on the central motor drive (Amann and Dempsey 2008; Amann et al. 2009). Previous studies have reported a decreased recruitment of fast-twitch skeletal muscle fibers during the last stage of maximal isokinetic exercise (Gerdle et al. 1989, Casey et al. 1996), thereby suggesting that even during maximal work the exercising muscle becomes increasingly more dependent on the supply of aerobic energy as the exercise proceeds. We would expect impairment in muscular function during isokinetic contraction in the hypoxia condition.

Kawahara et al. (2008), similarly, did not find any difference in muscular fatigue between normoxic and hypoxic situations, and the authors suggested that the low SpO_2_ in the hypoxic group was compensated by increased blood flow, as they found higher HR during the latter half of the hypoxic exercise. In our study, measured HR did not differ during the isokinetic test. Furthermore, these authors suggested that verbal encouragement given to the experimental volunteers at the start of the exercise may have affected the final outcome. In our study, we also gave verbal encouragement from beginning to end of the isokinetic exercise; this may also have affected the results of the isokinetic evaluation.

A change in the energetics pathways to supply the demand of the exercise may also have contributed to the lack of difference in muscle performance in the hypoxic condition. McLellan et al. (1990) found that a significantly larger increase occurs in the concentration of lactate in the muscle group exercise performed in the hypoxia compared to the normoxia group, suggesting that the inhalation of air with a low oxygen concentration can increase the rate of anaerobic glycolysis. It is possible that the isokinetic exercise performed by subjects during the hypoxic condition is predominantly anaerobic; in other words that the aerobic contribution is negligible.

## Conclusions

Unfortunately, the reasons for the differences between our data and those from previous studies that found a strength and fatigue decrement under hypoxic exercise conditions cannot be fully explained, but we believe that this may be due to our study employing a smaller exercising muscle mass that consumes a smaller amount of oxygen over a short exercise period. The disparity may also be attributable to a difference in the muscle group tested, velocity of muscular action, mode of contraction, the length of exposure to hypoxia, or intensity of hypoxemic stimuli. Collectively, our findings indicate that reduced arterial oxygenation *per se* has no effect on muscular isokinetic strength of the knee extensors.

## Abbreviations

FIO_2_: Fraction of inspired oxygen
HR: Heart rate
ICCs: Intraclass correlation coefficients
MVC: Maximal voluntary contraction
Nm: Newton-meters
PO_2_: Oxygen partial pressure
SpO_2_: Oxygen saturation.
